# Improvement of DBR routing protocol in underwater wireless sensor networks using fuzzy logic and bloom filter

**DOI:** 10.1371/journal.pone.0263418

**Published:** 2022-02-07

**Authors:** Hamed Karimi, Keyhan Khamforoosh, Vafa Maihami

**Affiliations:** Department of Computer Engineering, Sanandaj Branch, Islamic Azad University, Sanandaj, Iran; Hankuk University of Foreign Studies, REPUBLIC OF KOREA

## Abstract

Routing protocols for underwater wireless sensor networks (UWSN) and underwater Internet of Things (IoT_UWSN) networks have expanded significantly. DBR routing protocol is one of the most critical routing protocols in UWSNs. In this routing protocol, the energy consumption of the nodes, the rate of loss of sent packets, and the rate of drop of routing packets due to node shutdown have created significant challenges. For this purpose, in a new scenario called FB-DBR, clustering is performed, and fuzzy logic and bloom filter are used in each cluster’s new routing protocol in underwater wireless sensor networks. Due to the fuzzy nature of the parameters used in DBR, better results are obtained and bloom filters are used in routing tables to compensate for the deceleration. as the average number of accesses to routing table entries, dead nodes, Number of Packets Sent to Base Station (BS), Number of Packets Received at BS, Packet Dropped, and Remaining Energy has improved significantly.

## 1. Introduction

Wireless sensor networks have many land-based applications. Over the past several years, there has been a growing trend of using sensor networks in underwater environments and has attracted the attention of many researchers. The underwater wireless sensor network enables a wide range of water applications such as mine detection, distributed tactical monitoring, water quality monitoring, pollution monitoring, marine exploration, environmental monitoring, and accident prevention [1–3].

Underwater Wireless Sensor Networks (UWSN) use some of sensor nodes and base stations to be installed under the sea. These sensor nodes communicate through audio signals due to the long delay of radio signal transmission. A baseline scenario is that these sensor nodes send information to the base station (located at sea level) and from that base station. Underwater features provide distinct requirements for algorithms and protocols designed for underwater wireless sensor networks [4]. In three-dimensional space, wireless sensor networks are implemented underwater, in which case each node shows its behavior. In three-dimensional space, like two-dimensional space, nodes move data packets by sensor nodes from one place to another. 3D spaces use sensor nodes and single nodes. Sensor nodes are usually located underwater and in a specific area, and each sensor node sends the input data it receives to its nearest neighbor, and feedback to Sends the side of the sink node. The sink node can mainly be placed on the water or in the water and collects the packets sent to it and sends it to the base station (BS) [5, 6].

The signals used by wireless sensor networks are mainly acoustic radio signals and optical signals. But radio and optical signals are not used primarily in underwater wireless sensor networks and are commonly used in onshore networks. In addition, audio signals cover longer distances than radio and optical signals, even though they are as low frequency as radio signals. Therefore, it is consistent with the nature of underwater communications, which have a large Number of nodes together with the task of transmitting information at short distances, Therefore the signals used in underwater wireless sensor networks are mainly acoustic [7–10].

In underwater wireless sensor networks, the primarily role of routing protocols is to detect and maintain paths. Due to the different characteristics of underwater and environmental applications, terrestrial routing protocols are not suitable for underwater wireless sensor networks. Therefore, designing efficient, robust, and scalable routing protocols in underwater wireless sensor networks is challenging [11].

One of the most essential routing protocols in UWSN is the Depth Based Routing Protocol (DBR). DBR is a routing algorithm that attempts to send a data packet from one source node to multiple sinks [12, 13]. The DBR protocol involves the using a multi-sink architecture in which a number of underwater sensor nodes send information to the sink nodes. DBR only uses node depth information. The sensor node is equipped with a depth sensor for reaching the current depth node. The decision is based on the depth of the data and sends data packets from higher depth nodes to lower depth nodes. Normal nodes have more limited information than sink nodes, so DBR cannot transmit too much threshold (threshold means that normal nodes in DBR have restrictions on sending and receiving) [14]. Our comparative method in this paper is based on improved DBR or EEDBR [15, 16].

The fuzzy logic used in this paper is used as a theory to deal with most real-world phenomena in which there is uncertainty. In classical logic, membership in a set is considered zero and one. If there is a member in a set, it is denoted by 1; otherwise, it is marked by 0. The degree of membership is a function whose range is a member of the set [0 and 1]. But on the other hand, in fuzzy logic, the concept of degree of membership in a group expands to [0,1] [17].

In this paper, to improve memory, which ultimately leads to improved node power consumption, the DBR routing protocol uses a data structure called Bloom Filter, a standard proposed Bloom Filter implemented in NS-3 simulation.

The standard Bloom Filter is a random data structure introduced to represent a data set to reduce memory consumption. After saving the elements, this structure supports membership testing operations, so in cases where it is necessary to store a set of elements in a small space. The fault of Bloom filters is the small probability of an error that occurs when checking the membership of the query element, which is called a false positive. In this case, the query element that is not a member of the set stored in Bloom Filter is unlikely to be recognized as a set member [18, 19].

**Fig 1:**
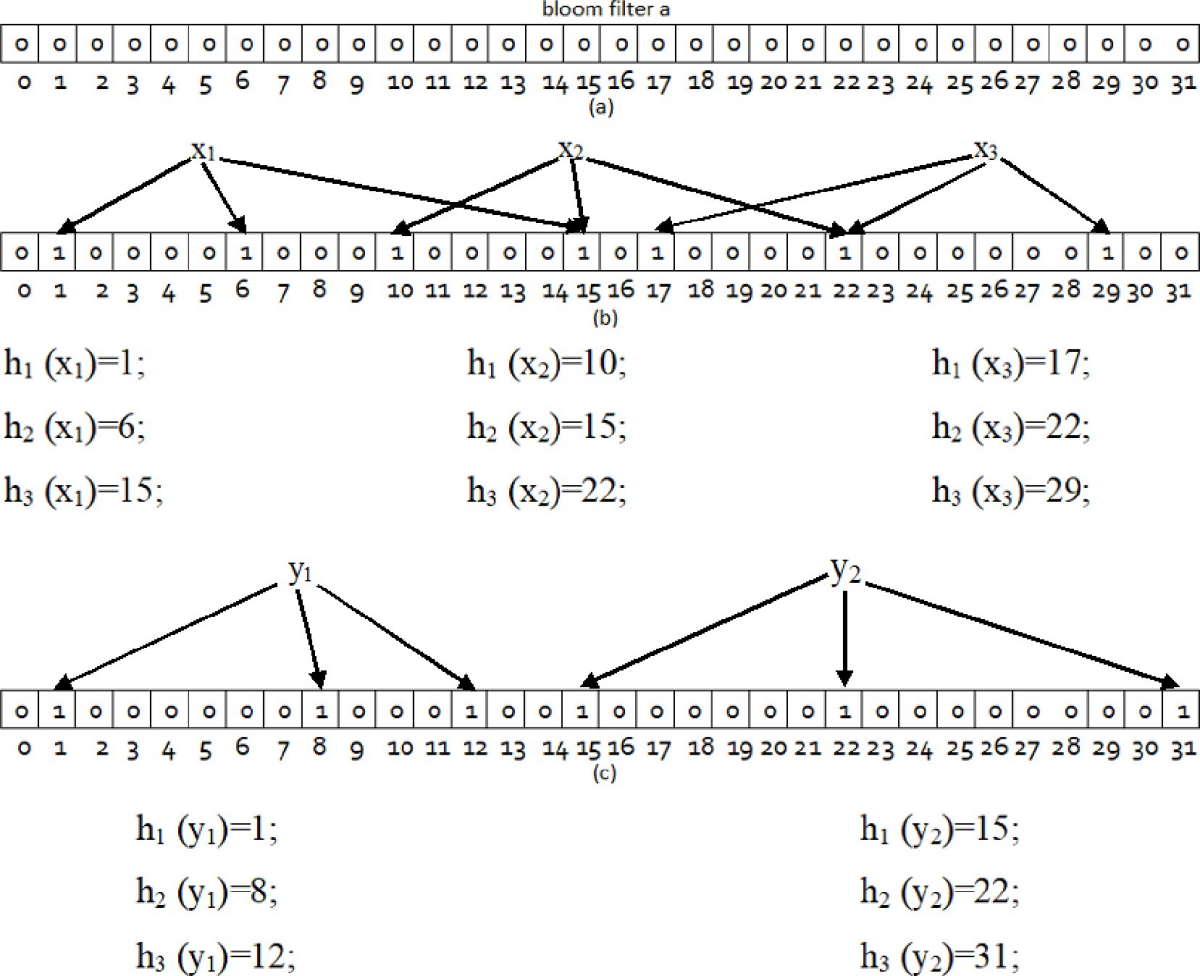
Insert element membership in bloom filter. (a) Initial bloom filter (b) Insert three elements *x*_1_ ،*x*_2_ and *x*_3_ (c) Query two elements *y*_1_ *and y*_2_.

Fig 1 shows the steps for making a bloom filter. Fig 1(A) shows the initial filter bloom in which all bits are set to zero. In Fig 1(B), the three independent elements *x*_1_ x_*2*_، and *x*_3_ are hashed separately by three independent functions, and The positions obtained in the bloom filter are set to 1. All k bits *A*[*h*_*i*_ (*y*_1_)] are checked for element *y*_1_ membership in Bloom Filter 1. According to Fig 1(C), since the twelfth bit has a zero value, we conclude that y1 is not a set member. But all three bits corresponding to the element *y2* have a value of one. This means that *y2* is a member of the set. Of course, in this case, with a very small error probability, this conclusion may be incorrect.

The following innovations are presented in this paper:

1. This algorithm is done by clustering and specifying routing destinies with a smaller number of methods—the role of node changes in different cycles for managing the energy of cluster head.
2. In this paper, fuzzy logic is also used to reduce energy consumption in DBR [9, 20]. Due to the fuzzy nature of the problem, parameters such as Energy Estimation, Expected Transmission Count, and hop count have been used as effective parameters in the result [21, 22].
3. Another innovation in the article is Bloom Filter, which uses a faster search of routing tables and thus improves the power consumption of nodes in DBR. Our goal in using Bloom Filter is to reduce the number of accesses to routing table entries, which ultimately speeds up routing, improves memory, reduces the number of packet-dropped, and ultimately energy consumption. The proposed bloom filter is standard [23–26].

## 2. Related works

In this paper, the main focus is on underwater routing protocols and IoT usage of these protocols. The term UWSN refers to underwater sensor networks, and IoT_UWSN refers to underwater Internet of Things networks. They are mainly used in data transmission in the DBR routing protocol. The first method is direct transmission so that each sensor node sends a data packet directly to the sink [27]. This condition may cause discharges that are too far from the sink node to drain [28, 29]. The second method of transferring data from the sensor node to the sink is hopped by hop. In this case, each node transmits data to the sink by selecting its nearest neighbor as the forwarding node, thus increasing a load of nodes closer to the sink node.

For this reason, at the very beginning of the network, their energy is quickly discharged and lost. The life of the network is reduced [30]. The third mode is known as Clustering-based routing. Each sensor node transmits the collected data to the corresponding clusters, and then each cluster sends its collected data to the base station [31–34]. In the third method, node-to-node transmission is minimized, bandwidth consumption is reduced, and network lifetime is increased.

Routing protocols for IoT_UWSN have sparked new research, such as the providing of smart cities. In different countries around the world, smart cities are becoming a reality. These cities increase residents’ satisfaction by providing regular help based on information obtained from wireless sensor networks (WSNs) and various components of the Internet of Things (IoT) [35, 36]. In the implementation of smart cities, applications such as smart ports and marine applications have been considered. The discussion of routing is an essential requirement for the implementation of these protocols. One of these protocols is the DBR routing protocol used in the field of smart cities.

In different researches, three scenarios with different base stations have been considered in UWSN. In the first scenario, a single sink node is located at the water surface. In the second scenario, the sink nodes are located at equal distances from each other on the surface of the water. In the third scenario, there are two categories of sink nodes. One of the sinks is located on the waters surface, and the other is in a three-dimensional space in the water in a predefined way. Each UWSN-based routing protocol and IoT_UWSN perform differently in each of the above three scenarios. In the proposed method, a combination of three different scenarios is used. In each of these scenarios, fuzzy logic and a bloom filter are used to improve the energy consumption of nodes in DBR.

Underwater Sensor Networks (UWSNs) have as of late been respected as promising to screen and investigate underwater situations. Reliable and productive information transmission to Sink is one of the foremost vital concerns of UWSNs. This paper proposes an Energy-Efficient Probabilistic Depth Based Routing for underwater sensor networks (EEPDBR in brief), moving forward from the conventional Depth Based Routing algorithm. The critical thought of the EEPDBR algorithm is to plan and make strides in probabilistic DBR algorithm for submerged information announcing to any surface sonobuoy. It takes the node’s profundity, residual vitality, and sending number within its 2-hop neighbourhood under consideration [37].

One of the productive approaches for data routing in underwater wireless sensor systems (UWSNs) is clustering. The information packets are exchanged from sensor nodes to the cluster head (CH). Data packets are at that point sent to a sink node in a single or numerous jumps conduct, which can increment the energy consumption of the CH as compared to other nodes. Whereas a few instruments have been proposed for cluster arrangement and CH determination to guarantee productive conveyance of data packets, less consideration has been given to gigantic information communication forms with sink nodes. As such, disappointment in communicating nodes would lead to a critical organize void-holes issue. Considering the constrained vitality assets of nodes in UWSNs and the overwhelming stack of CHs within the routing process, this paper proposes a void-holes aware and reliable data forwarding strategy (VHARDFS) in a proactive mode to control information parcels conveyance from CH nodes to the sink in UWSNs. Each CH node is mindful of its neighbour’s execution positioning file within the proposed technique to conduct a dependable packet transmission to the sink using the only energy-efficient course. Broad simulation shows that the VHARD-FS outperforms existing routing approaches whereas comparing energy effectiveness and arrange throughput. This makes a difference in effectively lightening the asset limitations related to UWSNs by expanding organized life and expanding benefit accessibility in a harsh underwater environment [38].

VBF (Vector-based Forwarding) is the first Geographically oriented routing protocol (known as modified DBR) designed for mobile underwater sensor networks by Xie et al. [39]. In VBF, data packets are routed along the routing vector from source to sink, and each node in the network knows its position. In VBF, when a sensor node receives a data packet, it first calculates its distance to the routing vector. If the distance from the predefined threshold is R. This node is a routing candidate and is eligible to send packets. VBF may use multiple simultaneous paths for improving reliability. It is not suitable for installing sporadic networks underwater and is sensitive to the threshold of the routing radius.

The DBR (Depth Based Routing) proposed by Yan et al. [40] uses a greedy method of delivering data packets to destination sinks at the surface of the water. In DBR, each node in the network does not require complete dimensional location information and only needs the local depth information of each node. An inexpensive depth sensor can easily measure depth information compared to complete location information. In DBR, sink nodes are used to collect data packets from underwater sensor nodes. In this algorithm, packets are forwarded from deeper sensor nodes to shallower sensor nodes [40, 41].

Baranidharan et al. say that the foremost unfavourable characteristics of underwater wireless sensor network (UWSN) communications are high propagation delay, high error rate, very low bandwidth, and limited available energy. The energy resources placement is additionally costlier. They proposed clustering-based geographic- astute routing with alteration of depth-based topology control for communication recuperation of void districts (C- GEDAR). The cluster-based GEDAR routes the parcel to the surface with the assistance of clusters. The void sensor node recuperation algorithm recuperates the void nodes from calculating their unused depth [42].

In [43], a modern algorithm named improved energy-balanced directing (IEBR) is outlined for UWSN. The algorithm incorporates two stages: steering foundation and information transmission. Amid the primary organize, a numerical show is built for transmission removal to discover the neighbours at the ideal separations, and the submerged organized joins are built up. In expansion, IEBR will select transfers based on the profundity of the neighbours, minimize the bounces in an interface and fathom the issue of the information transmission circle. Amid the moment stage, the joins built within the, to begin with arranging, are powerfully changed based on the energy level (EL) differences between the neighbouring nodes within the joins to attain the vitality adjust of the whole set and amplify the arrange lifetime altogether.

Challenges such as nodes’ mobility, increased propagation delay, limited bandwidth, packet duplication, void holes, and Doppler/multipath effect addressed the proposed paper entitled "An Efficient Routing Protocol based on Master-Slave Architecture for Underwater Wireless Sensor Network (ERPMSA-UWSN)". It significantly contributes to optimizing energy consumption and data packet’s long-term survival. Authors adopt an innovative approach based on the master-slave architecture, which limits the forwarders of the data packet by restricting the transmission through master nodes only. In this protocol, authors suppress nodes from data packet reception except the master nodes. We perform extensive simulations and demonstrate that our proposed protocol is delay-tolerant and energy-efficient [44].

## 3. Proposed method

**Fig 2:**
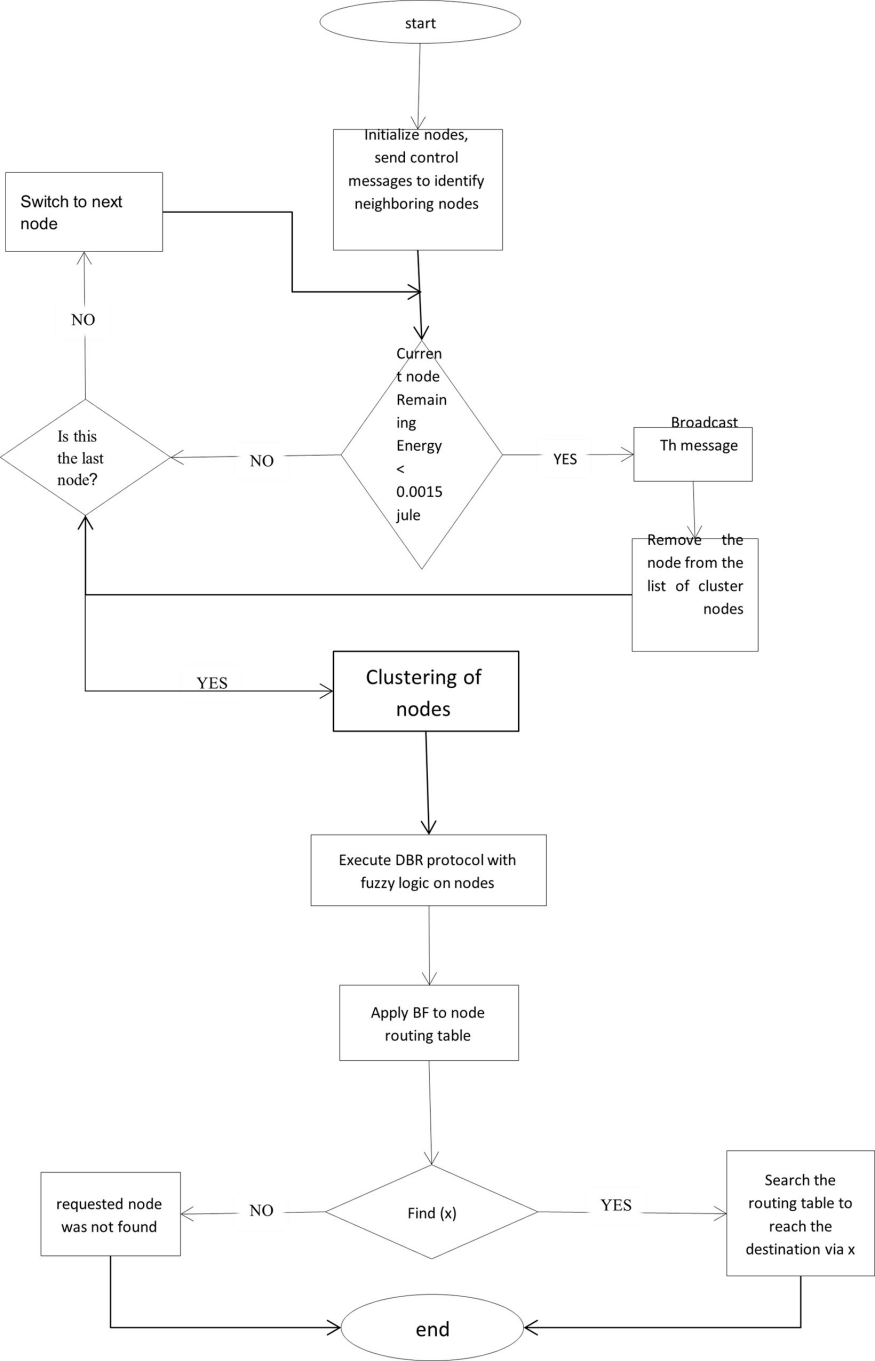
Flowchart of how the proposed method works (FB-DBR).

In this paper, a method for improving the algorithm is fully described in Section 2. First, for more accuracy, all nodes are divided into smaller clusters. Each clusterhead is responsible for routing packets to the nodes within the cluster and tries to be more accurate in routing by making fuzzy decisions to choose the best path. The proposed algorithm is presented in 4 different steps. The details are given in the flowchart of Fig 2. First, in step one, the nodes are initialized, and in step 2, clustering and selection of the clusterhead are made. In step 3, the DBR is modified with fuzzy logic to be more accurate in routing, and in step 4, a bloom filter will be used to improve the routing speed. Each of the four steps is described in detail below.

### Step 1: Initialize nodes, send control messages

In this step, the sink node sends a control message to other nodes in its range, and each node that receives this message puts its value in the Rank field and sends it to the nodes in its range. In this way, each node identifies its neighbor and calculates the distance from them.

### Step 2: Clustering

At this stage, cluster nodes are selected based on the remaining energy, and by calculating the threshold limit, the cluster nodes are selected [45, 46]. Any node with a higher threshold (i.e. above 0.0015 joules) is on the list of clusterhead candidates and can receive and send messages to the BS for the next round as clusterhead for that group. The formula for calculating the threshold is calculated from Eq (1):

$$f(n)=\left\{ \begin{array}{l} \frac{P}{1-P\;(r\;mod\;\frac{1}{P})}\;if\;n\in G\\ 0\;otherwise \end{array}\right.$$ (1)

In Eq 2, P is the optimal percentage for clusterheads. For example, P = 0.05 and r is the current stage, and G is the set of nodes that were not selected as clusterheads in 1/ p the last step. In stage 0, each node is clusterhead with the probability P They will not be clusterhead until the next stage of 1/p, so the probability of clusterhead other nodes increases.

Then the other remaining nodes are added to it based on the parameter close to the node, and by moving the control packets between node and clusterhead, a cluster is finally created. Eq 2 is used to calculate the probability of clusterhead based on distance. The positions of X and Y in the simulator are selected randomly.


$$prob\;(i)=\alpha \;\left(\frac{Remaining\;Energy}{Initial\;Energy}\right)+\beta (\frac{1}{d_{ij}})$$ (2)

In Eq 3, the selection of the cluster head based on the maximum energy remaining and the minimum distance is considered. In this relation, α and β are the equilibrium factors for the energy remaining and the distance between the nodes. As seen from the flowchart, clustering is repeated at different intervals to prevent it from remaining steady and consuming too much energy.

### Step 3: Apply DBR algorithm and fuzzy logic for routing

In this step, the DBR algorithm is applied to route the nodes of each cluster. In NS-3 simulation, this algorithm does not exist by default, and processing development in the simulator must implement this algorithm. The value of nodes is calculated using the fuzzy method to implement fuzzy logic and based on parameters of energy estimation, expected Transmission Count, and Hop Count to the BS node in the objective function [47, 48]. Network requesting node receives control messages and selects the best route to send its packets based on the Rank field comparison. How to choose the clusterheads in each cluster and how to place the nodes in the environment is specified according to [45, 46].

**Fig 3:**
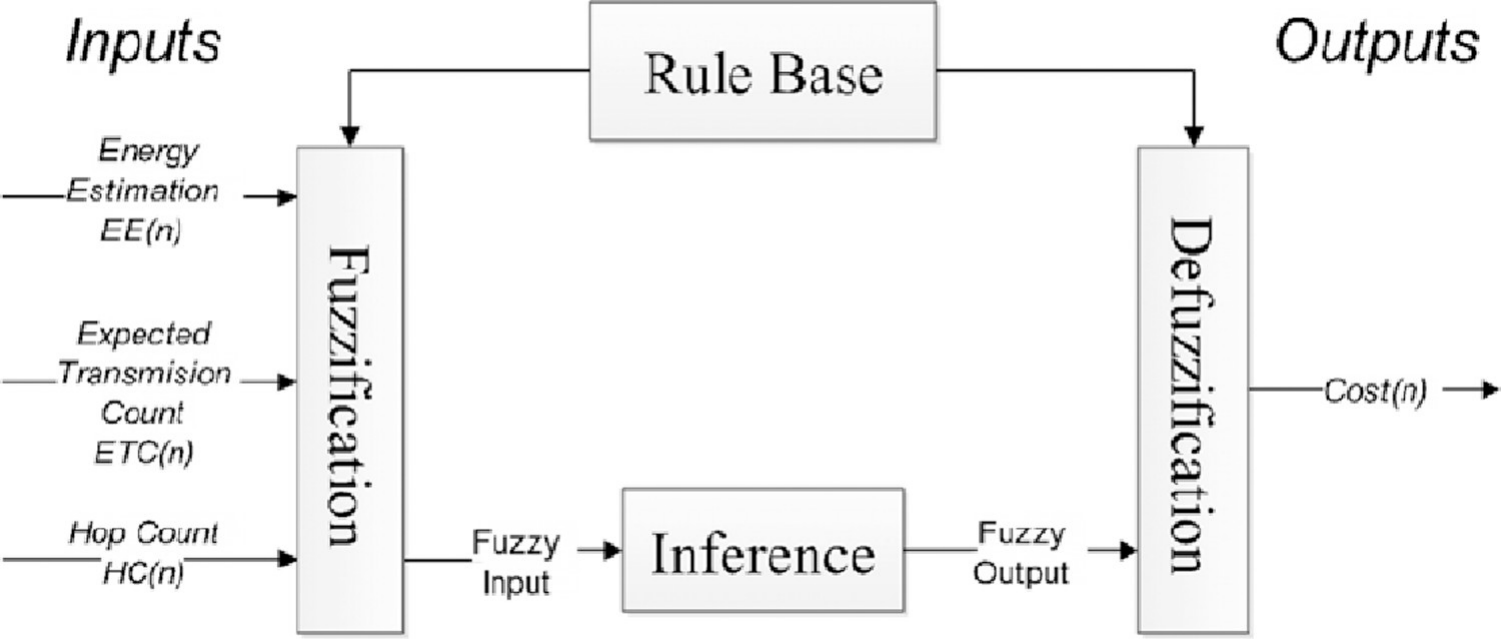
Schematic of the proposed fuzzy system.

Fig 3 shows the proposed fuzzy system structure. As shown in this Figure, the input values of the three values are indicated, and the output is the cost determined for each node’s selection in routing.

Each node in the network in each unit will have a specific position when it is taken from three parameters of energy estimation, expected transmission countand hop count to the root node. But these three parameters, after entering the proposed fuzzy system and performing fuzzy operations, and complying with the rules, become the output of the fuzzy system, which will be considered as the cost of the node or *NC*_(*n*)_ and is calculated from Eq (3):

$$NC(n)=\frac{\sum_{i=1}^{n}U_{i}\times C_{i}}{\sum_{i=1}^{n}U_{i}}$$ (3)

The *U*_*i*_ parameter will be the Cost estimate for node I, and *C*_*i*_ will be the actual cost for node i. Finally, the sensor node with the highest *NC*_(*n*)_ value can be selected as the most suitable clusterhead. For example, in each sensor node, a table is created according to Table 1, As shown in this Figure, the input values of the three values are indicated, and the output is the cost determined for each node’s selection in routing. updated after different time intervals.

**Table 1:** Parameters for each sensor node.

*Node ID*	*Remaining Energy (n)*	*Expected Transmission Count(n)*	*Hop Count(n)*	*NC*_(*n*)_
*9*	*0*.*019*	*0*.*45*	*5*	*0*.*32*
*17*	*0*.*021*	*0*.*58*	*4*	*0*.*39*
*46*	*0*.*025*	*0*.*54*	*2*	*0*.*48*
*81*	*0*.*017*	*0*.*58*	*3*	*0*.*44*

**Fig 4:**
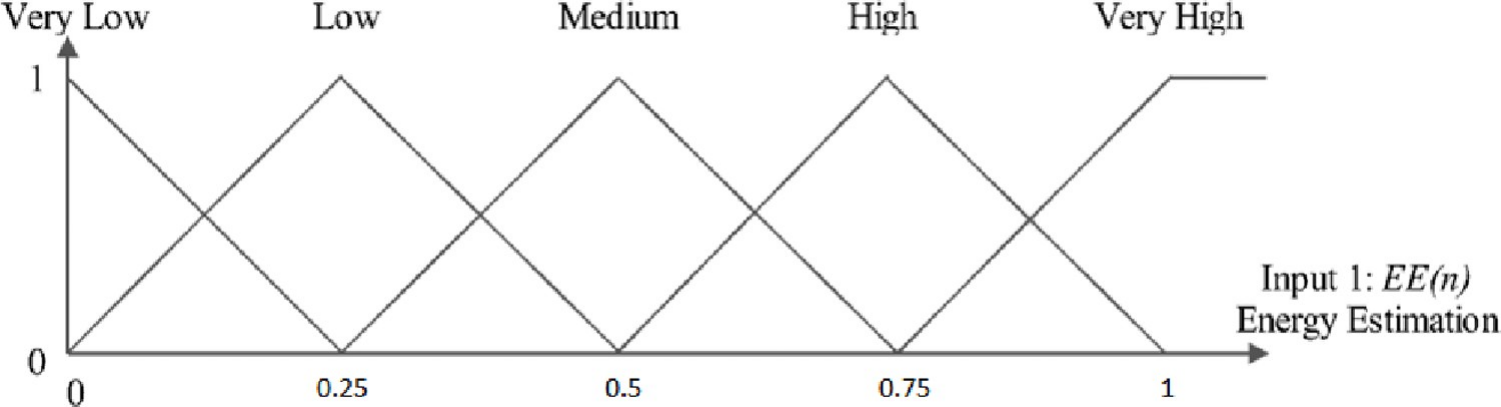
Graph of node energy estimation.

In this paper, fuzzy logic and triangular model are used. In each diagram, according to the specified triangles, other values in Y can be attributed to the behavior of a parameter in the X variable. Each point on the X-axis has two values on the Y-axis. Fig 4 shows the estimated energy of the node. At the beginning of the simulation, the initial energy of the nodes is 0.03 joules, and for the lowest state of the node energy, the value is considered zero. This energy estimate can be divided into five levels: Low, Very Low, Medium, High, Very High. The estimated energy of each network node can be at one of these levels or finally at two consecutive levels. The higher the estimated energy of the node, the more valuable the node.

Fig 4 shows the proposed three fuzzy logic inputs. These inputs include energy estimation, expected transmission count, and hop count. The higher this value for a node, the higher the priority is given to the selected node to select the most suitable clusterhead. In other words, increasing this parameter increases the company’s chances of choosing the most suitable clusterhead. Fig 4 shows an example based on the estimated energy.

**Fig 5:**
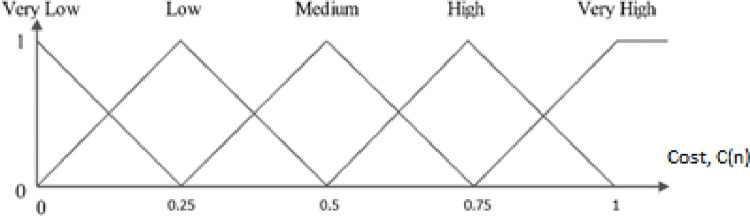
Fuzzy diagram of cost calculation for each node combination condition (cost function).

After applying Inference, the fuzzy output obtained from the applied inputs follows Fig 5, and the cost is fuzzy. Fig 5 shows the cost of each state of the node state in range [0,1]. The higher cost, the higher value of the proposed relationship.

**Fig 6:**
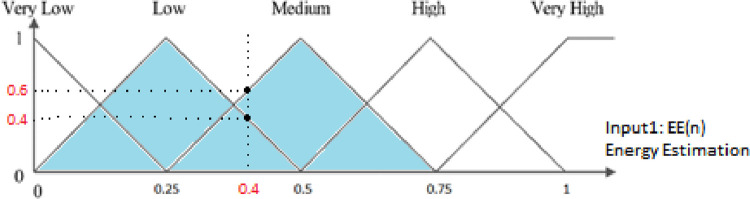
A numerical example of the estimated energy value of a node and its fuzzy outcome.

For example, an example of calculating the cost of a node is described. Assume that node A has an energy estimation of 0.4, an expected transmission count of 0.7, and a hop count of 6. According to Fig 6, node energy is located on the middle and bottom triangles. At point 0.4, draw a vertical line on X-axis. Intersects these two triangles in the middle and lower center. The cutting points are mapped on Y-axis, and the values of EE (low) and EE (medium) are 0.4 and 0.6, respectively.

By applying the same approach to the values of expected transmission count and hop counts, the results after defuzzification are obtained as follows:

EE = 0.4, Low&Medium→ L = 0.4, M = 0.6

ETC = 0.7, Medium&High→ M = 0.2, H = 0.8

HC = 0.6 Medium&High→ M = 0.7, H = 0.3

The output values for results obtained in this example are summarized in Table 2, the largest of which will be cost function value. In the table, the three levels used for fuzzy are multiplied.

**Table 2:** Showing each rule and its numerical value.

Rules	Value	Result
*EE*_*Low*_×*ETC*_*Medium*_×*HC*_*Medium*_	0.4×0.2×0.7	0.056
*EE*_*Low*_×*ETC*_*High*_×*HC*_*Medium*_	0.4×0.8×0.7	0.224
*EE*_*Low*_×*ETC*_*Medium*_×*HC*_*High*_	0.4×0.2×0.3	0.024
*EE*_*Low*_×*ETC*_*High*_×*HC*_*High*_	0.4×0.8×0.3	0.096
*EE*_*Medium*_×*ETC*_*Medium*_×*HC*_*Medium*_	0.6×0.2×0.7	0.084
*EE*_*Medium*_×*ETC*_*High*_×*HC*_*Medium*_	0.6×0.8×0.7	0.336
*EE*_*Medium*_×*ETC*_*Medium*_×*HC*_*High*_	0.6×0.2×0.3	0.036
*EE*_*Medium*_×*ETC*_*High*_×*HC*_*High*_	0.6×0.8×0.3	0.144

### Step 4: Apply bloom filter to the node routing table

Bloom filter must be used to send packages. BF specifies whether node to be sent depends on in routing table. If there is a node in the routing table, the BF array returns the value True at Time o (k) (K here is a fixed number and represents the number of keys used in BF), and then it must be returned in the routing table at time O(n) searched for the destination node. If the destination node is not present in the routing table, the BF array returns a False value at time O(k). In this case, there is no need to search for the destination node in the routing table. How to use BF is shown in the packet routing table follow.

In the proposed method, before sending a packet to another node, each node must pass that node to the Bloom filter before entering the routing table and specify whether this node is in the routing table. If the node is not in the routing table, it is no longer necessary to search in the routing table, and if this node is in the routing table, BF returns True, then it should be searched in the routing table. (Search in this simulation is sequential. Whose cost is of the order of O (n)).

**Fig 7:**
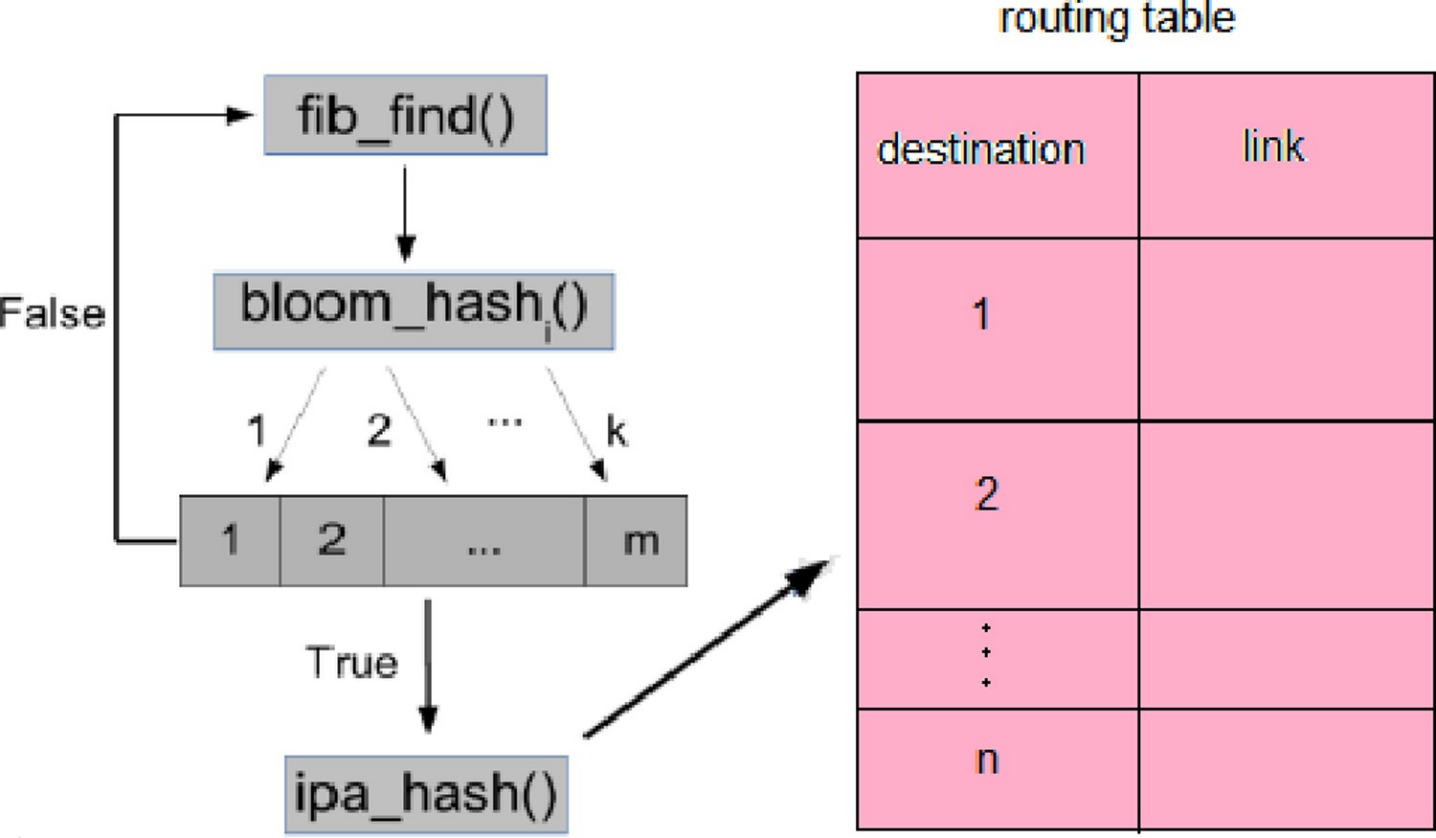
Proposed bloom filter system.

Fig 7 shows the proposed BF system. The main discussion in this method is to improve the nodes’ memory by reducing the number of accesses to entries in the routing table. The number of packets sent and received increases and even the amount of energy consumed by the nodes is reduced. In addition, hash functions are used before sending to the BF array and before sending to the routing table [49–52].

fib_find function is expressed in Fig 7 as Psuedo-Code1. This function searches the routing table and finds the node specified by the ipa_hash () function. If found, it returns node link otherwise returns null [53–57].

**Psuedo-Code1. fib_find()**

**fib_find(fib, prefix, length)**

**1. e = fib_table[ipa_hash(prefix)]**

**2. for(i = 1 to n)**

**3.    if (found e)**

**4.        return e**

**5.    Else**

**6.        Return null**

In the standard Bloom filter, we have the k hash search function, which is used to insert elements into the Bloom filter array. In pseudo-code 2, the bloom_hash () function first specifies whether the node in question exists in the BF array. If it does not exist, the function returns the value null, and if the node is found in the BF array, it returns the value e to the fib_table function to search for the node.

**Psuedo-Code2. BB_fib_find()**

**BB_fib_find(fib, prefix, length)**

**1. for(i = 1 to k)**

**2.    if(filter[bloom_hashi(prefix)] is empty)**

**3.        return NULL**

**4. e = fib_table[hash(prefix)]**

**5. for(i = 1 to n)**

**6.    if (found e)**

**7.        return e**

**8.    Else**

**9.        Return null**

Sometimes the prefix you are looking for may not be present in Bloom Filter, but the bloom_hash (prefix) function erroneously returns true, called a false positive. In pseudo-code 3, following the node in the hash array, it is first searched with length = k, then if the element is not found, it is subtracted from the length value of one unit until the desired element is found. Finally, if the node is not found, this function returns the null value. To set the value of k in the Bloom filter array, you need to look at the size of the routing table. For example, if the, value of k is assumed to be 2, in some cases, there may be less routing overhead than the value of 3. Therefore, it is better to set the value of k dynamically according to conditions.

**Psuedo-Code3. SB_fib_route()**

**SB_fib_route(fib, prefix, length)**

1. **while** (length ≥ 0)

2.    **if**(fib_find(fib, prefix, length))

3.        **return** found node

4.    **else**

5.        length = length– 1

6.    **return** NULL

## 4. Evaluate the proposed method

To evaluate the proposed method, the proposed FB-DBR protocol, which uses fuzzy logic and Bloom filter, as compared with the DBR protocol, which was simulated using NS3 simulator software version 3.25, and evaluated the results. Table 3 shows the simulation conditions and implementation assumptions.

**Table 3:** Simulation conditions.

UDP	Transmission protocol
DBR	Routing protocol
Drop-tail	Queue type
100× 100 × 100	Network size
Omni-Antenna	Antenna type
30min	Simulation time
0.03j	The average initial energy of the root node
End of network	Root node transmission range
Random	Position of nodes
0.03j	The average initial energy of the normal node
300m	Normal node transmission range
20	Queue length
Energy-Model	Energy model
CBR	The type of data generated
50 byte	Packet size
Fixed	Bit rate
18 Mbps	Transmission rate

In this paper, three parameters area, Number of nodes, and pause time are presented for evaluation. Each parameters takes different values, and the DBR routing protocol is compared with FB-DBR (proposed method).

### 1) Based on Area

**Fig 8:**
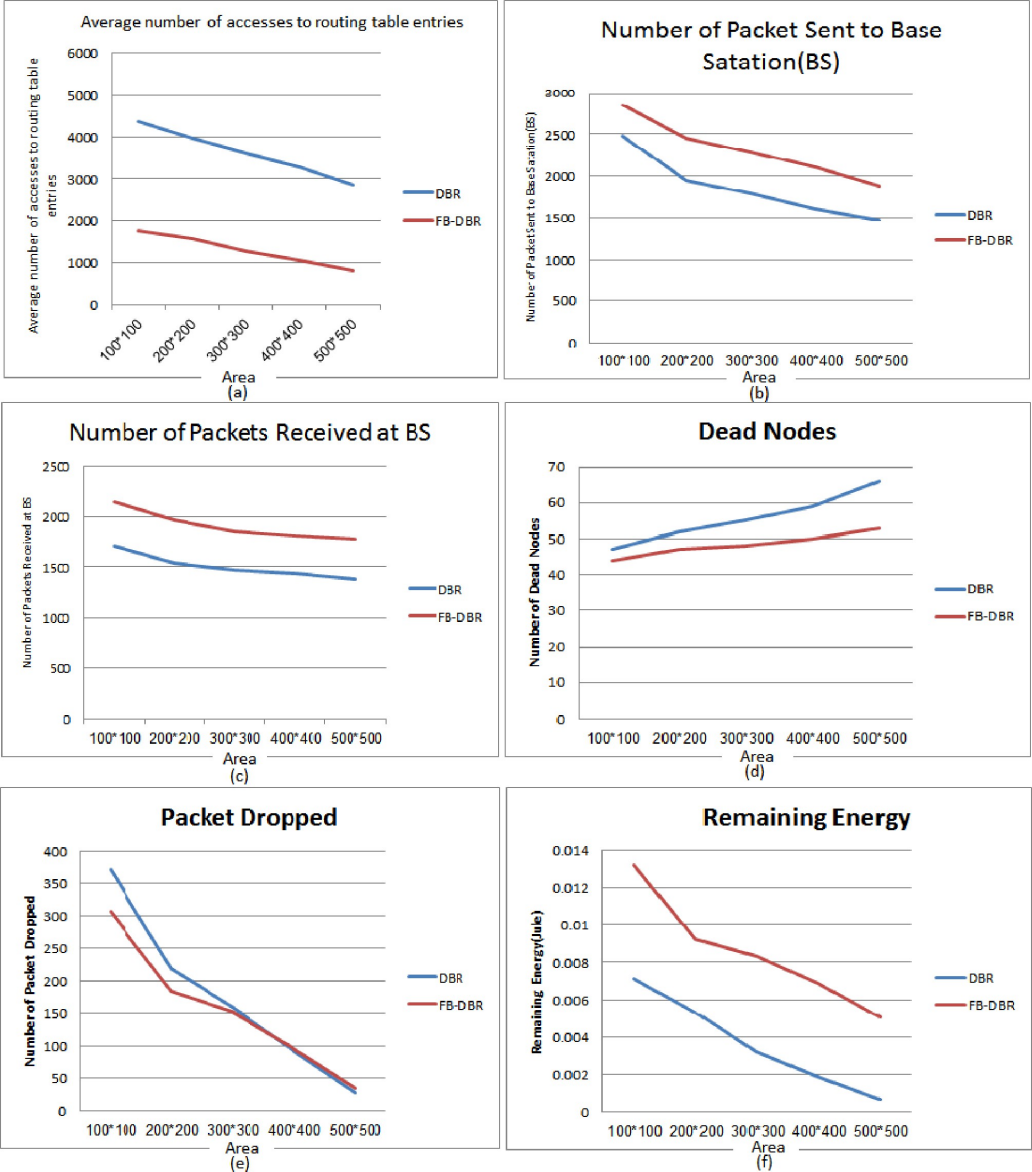
FB-DBR and DBR outputs based on Area.

Fig 8 compares six different parameters in FB-DBR with DBR based on Area, in most of which FB-DBR performed better than DBR. The simulation has areas of 50 × 50, 100 × 100, 200 × 200, and 400 × 400 square meters with 100 nodes and 30 minutes.

In Fig 8A, the average number of accesses to routing table entries is evaluated. Graphs are descending in both methods because the larger the simulation area, the greater number of packets loss and the greater number of masses loss. As can be seen, the proposed method provides less access to the tables due to the use of the Bloom filter in it. In Fig 8B, the Number of Packets Sent to the Base Station is evaluated. Graphs are descending in both methods because there are fewer packet losses in a small area than in higher areas. However, the proposed method shows better results. The highest number of packets sent to the workstation in the proposed method is due to fuzzy routing. In Fig 8C, the Number of Packets Received at BS is evaluated. Many packets are dropped in small areas due to the high volume of packets sent. While this issue is not raised in higher Areas, In Fig 8D, the Number of Dead Nodes is evaluated. The larger the simulation area, the higher the number of dead nodes, but in FB-DBR, due to the use of fuzzy logic and clustering of nodes, the energy consumption balance between the nodes is established, and the number of dead nodes is less. In Fig 8E, the Number of Packet Dropped is evaluated. FB-DBR in small environments has fewer packet drops than regular DBR due to the large volume of packets, but in larger areas, there is not much difference between the two. In Fig 8F, Remaining Energy (in terms of joules) is evaluated. Here we mean the average remaining energy of the nodes, which in all areas due to the use of fuzzy logic, Bloom filter, and clustering of nodes FB-DBR shows better performance than DBR.

### 2) Based on Number of nodes

**Fig 9:**
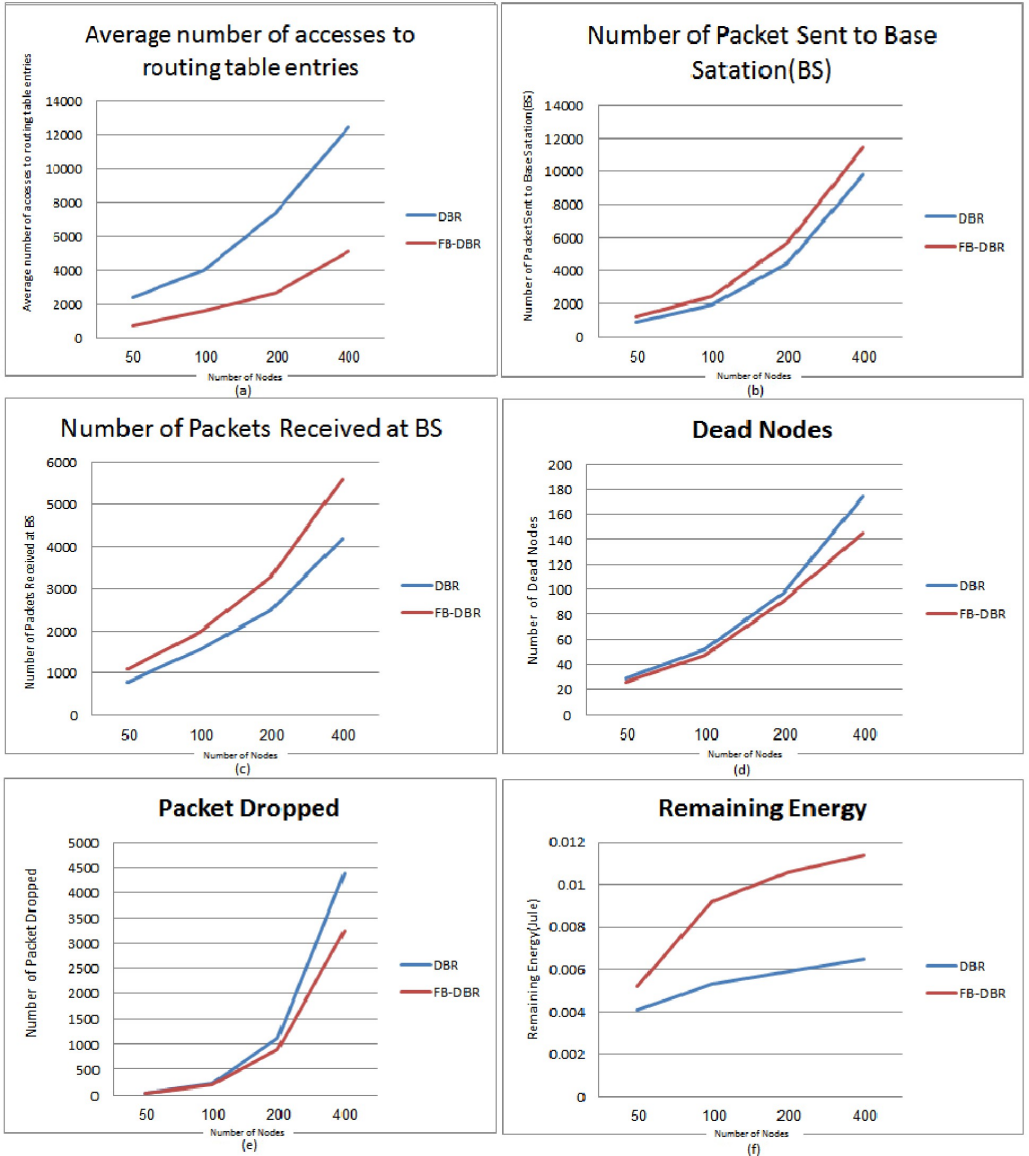
FB-DBR and DBR outputs based on the Number of Nodes.

In Fig 9, six FB-DBR evaluation parameters are compared with DBR in terms Number of nodes, in most of which the FB-DBR performed better than the DBR. Nodes in an area of 200 × 200 square meters with 50, 100, 200, and 400 nodes. Time of 30 minutes are implemented in the simulation. In Fig 9A, the average number of accesses to routing table entries is evaluated. Figure is ascending in both methods because the more nodes, the more packets are sent and received, and the more access to routing table. The Fig 9A shows that in all cases, especially when the Number of nodes increases, FB-DBR performs better than DBR. In Fig 9B, the Number of Packets Sent to the Base Station is evaluated. The figure is ascending in both methods because the more nodes, the more packets are sent and received. Fig 9B shows that in all cases, especially when the Number of nodes increases, FB-DBR performs better than DBR. This is due to the use of fuzzy logic and a Bloom filter in DBR. In Fig 9C, the Number of Packets Received at BS is evaluated based on the Number of nodes. The Figure is ascending as in the previous figures, and this is due to the increase in the Number of nodes. The delivery rate of routing packets in FB-DBR is shown to be higher than DBR. In Fig 9D, Number of Dead Nodes is evaluated based on the Number of Nodes. The Number of dead nodes in the case of increasing the Number of nodes in the FB-DBR method offers better performance than DBR. In Fig 9E, the Number of Packet Dropped is evaluated based on the Number of Nodes. The Number of packets dropped in the case of fewer nodes is not much different in both methods, but the higher the Number of nodes in the simulation, the better FB-DBR performs than DBR. In Fig 9F, Remaining Energy (in terms of joules) is evaluated based on the Number of nodes. The following Figure shows that when the Number of nodes is less, the nodes consume more energy due to more packets being lost. The following Figure shows that when the Number of nodes is minor, more packets are lost; the nodes consume more energy. With the higher Number of nodes, due to the proximity of nodes to each other, the use of Fuzzy logic, bloom filter, and clustering of nodes in the proposed method, the energy consumption of nodes is also reduced.

### 3) Based on Pause Time

**Fig 10:**
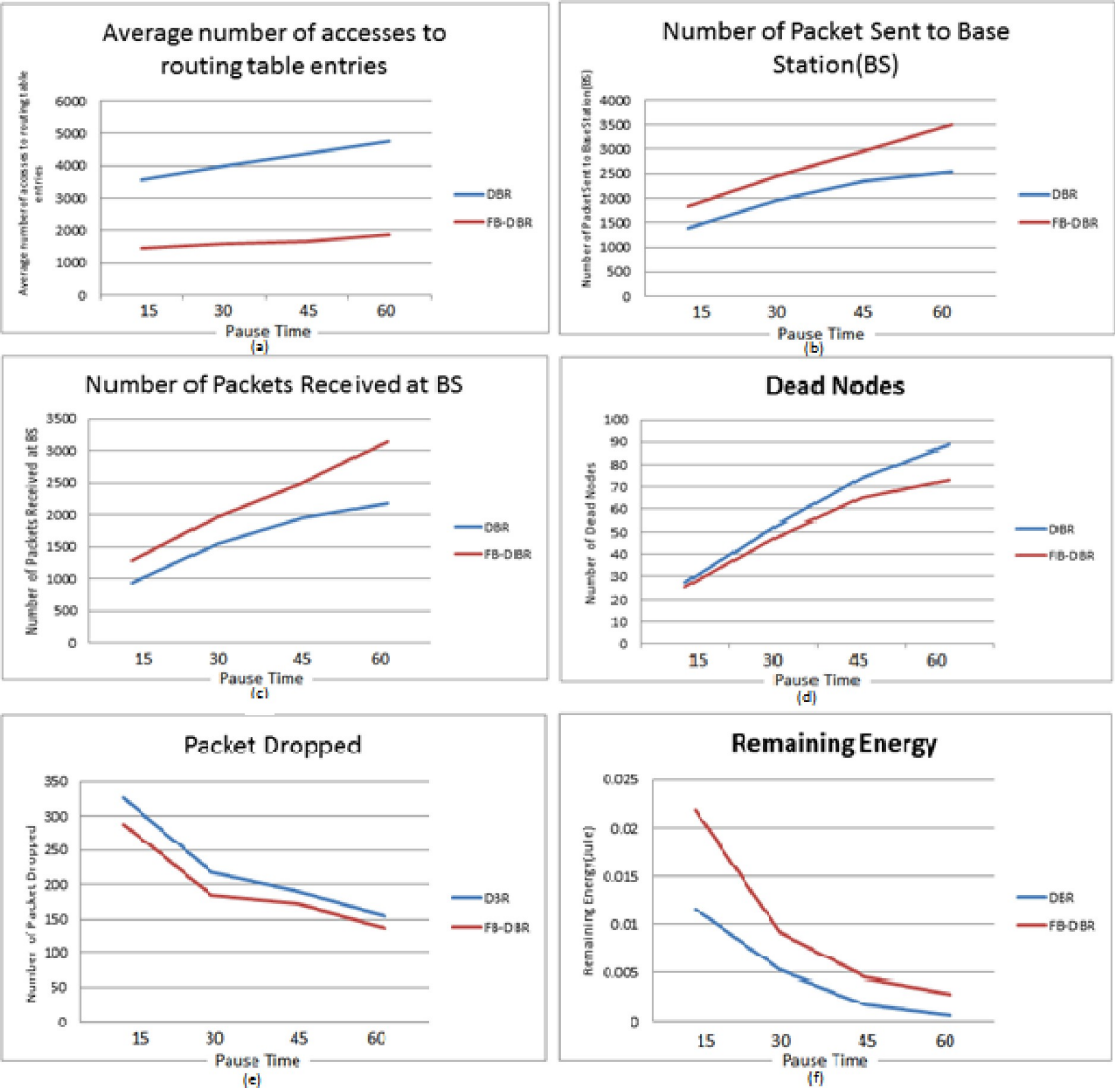
FB-DBR and DBR outputs based on Pause Time.

In Fig 10, six evaluation parameters of FB-DBR are compared with DBR based on runtime, in most of which FB-DBR performed better than DBR. The nodes are simulated in an area of 200 × 200 meters with 100 nodes, and a Pause Time of 15, 30, 45, and 60 minutes. In Fig 10A, the Average number of accesses to routing table entries is evaluated based on Pause Time. The Figure performs uniformly in both methods, indicating that FB-DBR is not time-dependent and performs better than DBR whenever the simulation lasts. In Fig 10B, the Number of Packets Sent to Base Station is evaluated based on Pause Time. The Figure shows that the number of packets sent in FB-DBR is more than DBR, and the longer the simulation time, the greater difference between the two. Because in DBR, number of dead nodes also increases with increasing simulation time. In Fig 10C, Number of Packets Received at BS is evaluated based on Pause Time. The Fig 10C is similar to Fig 10B, meaning that the longer the simulation time, the better FB-DBR performs compared to DBR. In Fig 10D, the Number of Dead Nodes is evaluated based on Pause Time. In this Figure, the longer the simulation time, the deader nodes in DBR than in FB-DBR. In Fig 10E, the Number of Packets Dropped is evaluated based on Pause Time. The Number of Packet Dropped has a downward trend because the Number of dead nodes is less in fewer simulation times, so the Number of Packet Dropped is higher. In Fig 10F, Remaining Energy (in joules) is evaluated based on Pause Time. The longer the simulation time, the lower the Remaining Energy of the nodes, so that the number of dead nodes at longer simulation times increases.

## 5. Conclusion

Nowadays, underwater networks and the discussion of their routing have become very important due to their various applications. In this paper, a method to improve the DBR algorithm is proposed. First, for more accuracy, the whole nodes are divided into smaller clusters. Then each clusterhead is responsible for delivering the routing to nodes inside cluster and tries to be more accurate in routing by making fuzzy decisions to choose the best route. Bloom filters have also been used to improve routing speed. Choosing the best route depends on various parameters, many of which are fuzzy.

In this paper, first the clustering is done, then the DBR algorithm performs routing based on fuzzy logic with the parameters of Energy Estimation, Expected Transmission Count and Hop Count. Enlarging routing tables uses a bloom filter, which greatly speeds up routing. Output based on parameters such as dead node, average number of accesses to the routing table entries, number of packets received in BS, remaining energy and packet dropped show a significant improvement in the proposed method compared to DBR. Each of these comparisons with different areas, number of nodes and stop times is examined and the improvement results are confirmed in the proposed method.

Output based on dead node parameters, average number of accesses to the router table, number of packets received in BS, residual energy and missing packets show a significant improvement in the proposed method compared to DBR. As a suggestion for future work, this idea can be applied to other routing protocols such as RPL routing protocol in IoT and routing protocols such as AODV and DSR. Also applied and redesigned them fuzzily.

## Supporting information

S1 File (ZIP)
